# On the Treatment of Colica Pictonum by Alum, under the Direction of M. Kapeler, Physician in Chief of l'Hôpital Saint Antoine

**Published:** 1829-02

**Authors:** M. D. Montanceix


### COLICA PICTONUM.

*On the Treatment of Colica Pictonum by Alum, under the direction of* M. Kapeler, Physician in chief of l'Hôpital Saint Antoine. By M. D. Montanceix.

For the last thirteen years, M. Kapeler has treated colica pictonum with alum, with a very favorable result. From fifteen to twenty persons affected with this disease are annually received into the hospital. The practice adopted at La Charité, which consists of drastic purgatives, sudorifics, and narcotics combined, is that which is usually had recourse to in France. As a proof of the efficacy of M. K.'s treatment, the following interesting cases are related.

Case I.—L. Bouligny, of a good constitution, aged nineteen years, a house painter by trade, was admitted on the 20th of February. The symptoms were as follow: For the last eight days he had been obstinately constipated, notwithstanding several clysters had been given. Great pain in the abdomen, which was rather relieved by pressure. Gnawing sensation of the stomach. Tongue dry and white; mouth bitter; urine scanty. Pulse forty in a minute; no headache. He has had no sleep for the last four days.

On the day of his admission, he took a mucilaginous mixture, with a drachm of the sulphate of alum, a tablespoonful each hour. An emollient clyster was administered. Barley and linseed water for common drink; spare diet.

21st.—Pulse quicker; tongue not so dry; less bitterness of the mouth; pains diminished; two hours' sleep. He has had two motions in the night, and has made water three times.

The same remedies continued.

In the evening, all the abdominal symptoms had ceased, and the pulse was nearly natural.

22d.—Continues better.

To take half a drachm of the sulphate of alum in the mixture as before. Broth diet.

25th.—Perfectly free from all symptoms of the disease.

26th.—Complains of pain in the head, with symptoms of general excitement. He was bled, and a blister was applied to the neck; and soon after he left the hospital.

Case II.—C. Baudin, an earthenware potter, of a weak constitution and lymphatic temperament, aged thirty-one years, was attacked, on the 26th February, with very acute pains in the belly, which obliged him to roll upon the ground. He uttered the most piercing cries, and was thrown into various positions. His suffering was somewhat relieved by applying a tight bandage round the belly. He was sleepless, complained of headach, and had been constipated for two days. He had a creeping sensation over all his limbs.

27th.—He was admitted in the following state: Extreme depression, trembling, and convulsive movements of the upper limbs; cramps in the lower limbs. The eyes were particularly brilliant. Acute pains in the abdomen, which were relieved by pressure, although the patient was averse to it. Retraction of the belly. Tongue dry, and of a blackish colour. Urine of a small quantity, and red; bowels constipated. Pulse small, and thirty-five in a minute. He was delirious two hours after his admission, and the strait-waistcoat was required.

Ordered to have barley and linseed water for common drink. Mucilaginous mixture, with a drachm of the sulphate of alum. An emollient clyster was given. Strict diet.

28th.—In the same state. Delirium has been very violent during the night.

Sulphate of alum two drachms. An oily clyster every half hour.

In the afternoon, the patient had recovered his senses, and complained only of a slight pain in the epigastric region and in the head. His bowels had been freely open, and he had made a large quantity of water.

Sulphate of alum one drachm. Same diet.

He now became convalescent, and in eighteen days was discharged well.

Case III.—J. Maiseau, of a strong constitution and bilious temperament, forty years old, a cooper by trade, was admitted on the 27th February, in a state resembling intoxication. He was violently delirious; his look was wild, and he was apprehensive that some injury would be inflicted upon him. He was furious when his belly was pressed, but still the pain which he complained of appeared to be relieved. Pulse very slow.

The case was at first doubtful, but it was soon ascertained that he had several times been attacked with metallic colic, and had once been in La Charité for three months with this disease. He was ordered a drachm of the sulphate of alum and a purgative clyster. In three hours he was much calmer, and he had a tranquil night. No evacuation from the bowels.

28th.—Patient calmer, but still he had not regained his mental faculties. Pulse very slow; abdomen painful; continual shaking of the head; tongue dry and rough.

To take two drachms of the sulphate of alum.* Purgative clyster every hour. Linseed tea for drink.

In the evening, the patient had recovered his senses, but had no recollection of what had passed. Still pains in the belly. He is now amaurotic, and has entirely lost his sight. Trembling in all the limbs. Bowels have not been opened.

Sulphate of alum two drachms. Two purgative enemas.

29th.—Pain has ceased; no trembling; return of appetite. Continues amaurotic. Has had four motions in the night.

Repeat the same remedies.

March 1st.—No alteration.

2d.—Begins to distinguish objects.

Medicines continued. Allowed soup.

15th.—Sight entirely recovered.

From the 3d to the 12th, he has taken each day a drachm of the sulphate of alum. Several boils have appeared in various parts. He was discharged cured, after having been fifty-three days in the hospital.

Case IV.—J. Legrand, of an ordinary constitution and bilious temperament, forty-two years old, a lapidary by trade, has been treated fourteen times in the space of twenty years for colica pictonum. The first attack occurred when he was about twenty-one, six months after he commenced his business. He had always been admitted into La Charité. When he was first seen, on the 29th February, he said that, eleven days before, he was suddenly seized with general uneasiness and headach, which lasted four days. On the 25th, he had cramps in the lower extremities, and pains in the belly, which were at intervals very severe. He does not complain on pressure. Loss of appetite: nausea, vomiting. Convulsive movements of the arms. Tongue white and moist; bitter taste in the mouth; severe pain in the belly. Has been constipated for three days. Pulse forty.

Ordered, linseed and barley water for common drink. Mixture with gum and a drachm of the sulphate of alum. Purgative clyster. Strict diet.

In the evening, symptoms increased. Movements of the arms

* The precise mode in which this medicine was taken is not mentioned. We presume it was given in a mixture in divided doses, as is prescribed in the first case.—Editors.

frequent; powerful cramps in the legs; excruciating pain in the belly; bowels continue constipated; tenesmus.

To take sulphate of alum two drachms; two purgative clysters. Six drops of croton oil to be rubbed around the navel.

March 1st.—In the same state. Bowels not open.

Sulphate of alum two drachms. Two oily injections.

In the evening, still the same.

To take two drachms more of the alum.

2d.—No alteration. M. Kapeler, still confiding in the medicine, which had never disappointed him, prescribed again two drachms of the sulphate of alum, and three oily clysters.

3d.—Much relieved. Has had four motions in the night, and made water freely. Pulse nearly natural; he says he is well.

Ordered one drachm of the sulphate of alum, and broth.

5th.—Free from complaint.

Case V.—L. Felix, of a bilious temperament and weak constitution, aged eighteen, a house painter by trade, was admitted the 19th April. He had been attacked with colica pictonum five days ago. The disease was well defined: acute pains in the abdomen, which were neither increased nor diminished on pressure; bowels constipated; tenesmus; retraction and hardness of the belly; nausea and vomiting of greenish and filmy matter; tightness in the præcordia; features of the face altered; tongue dry and foul; pulse slow; headache; paroxysms of dyspnœa. All these symptoms yielded to a drachm of the sulphate of alum, and to a purgative enema, as to a charm. He had eight stools in the night.

The same treatment was continued for three days, and he was discharged perfectly cured.

Case VI.—P. Racine, of a strong constitution, and of a bilious and sanguineous temperament, aged forty-five, a house painter, had suffered from the disease seven times.

Symptoms: May 1st.—Slight pains in the hypogastrium; loss of appetite; tongue dry and white; breath fetid; headach; numbness of the arms, principally the right; loss of recollection of nouns and numbers; pulse slow; constipated bowels; urine acrid, and in small quantity. These symptoms had existed for six days.

Ordered one drachm of the sulphate of alum in a mucilaginous mixture, and a purgative enema. Linseed tea for beverage.

June 1st—Has had five or six motions during the night, and declares that he is cured. The pains, numbness, and headach are removed. He cannot count up to five.

Continue medicines. Broth diet.

2d.—Continues better.

3d.—Memory perfect. Discharged cured on the 7th.

Case VII.—P. Mahille, of a weak constitution and bilious temperament, twenty-three years of age, a house painter, had been twice attacked with colica pictonum. He had now been discharged one fortnight from La Charité, with every appearance of being permanently cured. He had avoided, since, the exciting causes of the disease; and still, eight days afterwards, it returned with more severity than ever. He was admitted into the Hôpital St. Antoine, July 5th.

Symptoms: Extreme depression of mind and body; dilated pupils; abdomen retracted, and *very painful* on pressure; continual and violent colic; no stool for two days; frequent nausea, no vomiting; wandering pains and creeping sensation in the arms; spasms of the lower extremities; headach; tongue dry, and rather black; bitter taste in the mouth; pulse thirty-two; skin cold and moist.

Ordered a mixture with gum, one drachm of the sulphate of alum. Purgative enema.

6th.—No motion. Pulse thirty; vomiting of greenish matter.

To take two drachms of the sulphate of alum; two oily clysters.

7th.—No alteration. No motion.

To take three drachms of the sulphate of alum, and to have an oily injection every half hour.

8th.—Colic diminished; pulse thirty-five. Still no evacuation from the bowels.

Medicines continued. Three drachms of alum repeated in the evening.

9th.—Had a copious motion in the night, and was immediately relieved. Pulse forty; tongue moist and white.

Remedies as before.

10th.—Has had two motions this morning.

Continued to take three drachms of the sulphate of alum till the 13th. On the 16th, discharged well.

Case VIII.—J. Roblin, of a strong constitution and nervous temperament, forty-six years of age, by trade a brazier, was attacked, the 20th July, with violent colic, which obliged him to roll upon the ground. The belly was surrounded by a towel, very tightly bound. He had drank milk freely, and used several clysters, to no purpose. The disease increased in severity, and on the 23d the following symptoms were present: Face pale and stupid; headach; loss of appetite; tongue white and moist; bitter taste in the mouth; dragging sensation in the stomach; pain around the navel, relieved by pressure; retraction of the belly; spasms of the arms; urine scanty, bowels constipated; pulse thirty-nine; skin cold and dry.

Ordered barley water for common drink; mixture with gum; sulphate of alum one drachm; purgative enema.

24th.—Better. Has had three motions in the night; urine copious in quantity. Pulse forty-six; belly still painful; numbness in the legs; spasms of the arms have ceased.

Remedies continued.

25th.—No pain in the belly; numbness of legs diminished; pulse fifty-five. Two motions.—To continue medicines.

26th.—Convalescent. Discharged July 2d, cured.

Case IX.—N. Dureux, of ordinary constitution and bilious temperament, fifty-one years old, a brass founder, felt, on the 18th July, slight colic, and an itching and creeping in the limbs; in the evening, trembling of the arms. He continued to work till the 24th; but, as the disease now became more severe, he was admitted into the hospital.

Symptoms: Face pale and anxious; tongue covered with a yellow mucus; bitter taste in the mouth; no appetité; excessive pain in the belly, particularly in the hypochondria; no increase of pain on pressure; heartburn, nausea, and vomiting of a greenish matter; urine scanty, bowels constipated; great debility; spasms of the limbs. Has passed a sleepless night. Pulse very slow.

Ordered, linseed tea and barley water for drink. Mixture with gum; one drachm of sulphate of alum. Purgative clyster.

27th.—Greatly improved. Pulse nearly natural; all the symptoms diminished in severity. Has had three motions, and an abundant discharge of urine. He afterwards slept very comfortably.

28th.—No complaint. Appetite good.

To take half a drachm of alum.

Discharged, the tenth day, well.

Case X.—P. Fournier, forty-five years of age, house painter, of a strong constitution and sanguine temperament, has suffered eight attacks of metallic colic. He has always been treated at La Charité, and has generally left that hospital with paralysis of the left wrist. For the last attack, he was six weeks in the Hôtel Dieu, after having been two months in La Charité. Since the first occurrence of the disease, he has almost always felt slight colicky pains; his bowels have been alternately constipated and relaxed; in short, his health has never been completely established.

September 22d.—Fifteen days ago, after his work, he was attacked with rather violent colic and purging; weakness in the limbs; loss of appetite, nausea, no vomiting, has continued, with varying symptoms, until the present time. The symptoms that now exist are obstinate constipation, tenesmus, and weight at the fundament; giddiness; scanty urine, of a red colour, and with very copious sediment; violent colic, which is neither increased nor relieved by pressure; extreme weakness; ardent thirst; tongue white and moist, mouth foul. Has had no sleep for five nights. Cramps and spasms in the limbs, principally on the left side. The left forearm was yesterday so senseless that, when it was placed near a powerful fire, he did not feel it. Pulse thirty; skin cold and dry.

Ordered, linseed tea for drink. Mixture with gum; one drachm of sulphate of alum. An emollient clyster.

23d.—Much better. Pulse 120; all the symptoms milder; has had some sleep. Bowels opened eight times one hour after the medicine; copious discharge of urine.

To continue the remedies.

24th.—Complains only of slight weakness in the left side, which was yesterday quite benumbed. Appetite good. Has had no sleep in the night; bowels been open twice; frequent inclination to make water.

Medicines to be repeated. Broth diet.

25th.—Improved in every respect. He has walked in the ward, and amused himself with reading. Still complains of want of sleep.

To take half a drachm of alum. Broth diet, and a little soup.

26th.—Free from pain. Cramp of the left arm, which is no longer benumbed. He cannot walk up stairs. Has had one motion; no sleep.

Remedies as before. A mild anodyne pill. A little meat allowed.

27th.—Better in every respect. Still want of sleep.

28th.—Well. Has slept all night. Has regained his strength.

After a detail of these cases, it is presumed, by M. Montanceix, that the efficacy of the practice of the Hôpital St. Antoine must be established. Thirteen years' experience have proved the value of the proposed remedy, which is both innocent and effectual.

It is to be observed, that the subject of the third case had only left La Charité seventeen days, when the disease returned with the most alarming symptoms, which were quickly removed by a few drachms of the sulphate of alum. In the seventh case also a relapse occurred, without fresh exposure to the original cause of the malady, after the patient had been discharged eight days from La Charité. Case the tenth requires but little comment: it clearly proves the efficacy of the treatment proposed.

Neither inflammation of the stomach or bowels has ever occurred. In most cases, three or four drachms of the alum were sufficient to render the patient convalescent, and in no one instance has a relapse happened. M. Montanceix has seen the patients frequently after their discharge, and is therefore able to speak confidently upon this point.

It will be perceived that the requisite dose of the remedy is not always in proportion to the severity of the disease. Cases, which commenced with very alarming symptoms, yielded to two or three drachms; others, which appeared mild in their character, resisted even eight or ten drachms. The physician must therefore act according to circumstances, but the first dose should not be more than one drachm.

The sulphate of alum is asserted to be the best remedy we possess against the metallic colic. We think, observes M. Montanceix, that it will entirely supersede every other medicine, if its effects are impartially compared with other modes of cure.

Alum has been before much extolled as a remedy in colica pictonum, although it has been of late but seldom used in this country. If it should be found, as is stated in the above interesting paper, that a relapse never occurs after its employment, it should certainly not be neglected. Dr. Grashuys considered alum as a specific in this disease.* Dr. Percival gave it in doses of fifteen grains every four or six hours, and he declares with unvarying advantage.† The same plan of treatment has also been highly recommended by the German physicians.—Editors.

* Tentam. de Colica Pictonum, et App.

† Obs. and Exp. on the Poison of Lead. 1767.

